# Does insulin resistance increase thyroid volume in patients with polycystic ovary syndrome?

**DOI:** 10.1590/2359-3997000000225

**Published:** 2016-11-07

**Authors:** Murat Sahin, Didem Demircioglu, Ayten Oguz, Dilek Tuzun, Mehmet Akif Sarica, Elif Inanc, Kamile Gul

**Affiliations:** 1 Kahramanmaras Sutcu Imam University Faculty of Medicine Kahramanmaras Turkey Kahramanmaras Sutcu Imam University, Faculty of Medicine, Department of Endocrinology and Metabolism, Kahramanmaras, Turkey; 2 Kahramanmaras Sutcu Imam University Faculty of Medicine Kahramanmaras Turkey Kahramanmaras Sutcu Imam University, Faculty of Medicine, Department of Internal Medicine, Kahramanmaras, Turkey; 3 Kahramanmaras Sutcu Imam University Faculty of Medicine Kahramanmaras Turkey Kahramanmaras Sutcu Imam University, Faculty of Medicine, Department of Radiodiagnostic, Kahramanmaras, Turkey

**Keywords:** Polycystic ovarian syndrome, thyroid volume, insulin resistance, luteinizing hormone

## Abstract

**Objective:**

To investigate the effect of gonadotropin, sex hormone levels and insulin resistance (IR) on thyroid functions and thyroid volume (TV) in polycystic ovary syndrome (PCOS).

**Subjects and methods:**

69 new diagnosed PCOS patients (age 24.82 ± 6.17) and 56 healthy control female (age 26.69 ± 5.25) were involved to the study. Fasting plasma glucose, lipid profile, insulin, thyroid stimulating hormone (TSH), free thyroxine (fT4), estradiol (E2), luteinizing hormone (LH), follicle stimulating hormone levels and urine iodine were measured in all participants. Thyroid and pelvic ultrasound were performed in all participants.

**Results:**

Insulin, HOMA-IR, LH, E2 and TV were higher in PCOS group (p < 0.05). TV was significantly higher in PCOS patients with IR compared to non-IR PCOS patients (p < 0.001), while TSH, fT4, and urine iodine levels were similar between these groups (p > 0.05). There was a negative correlation between E2 and TSH (p < 0.05) and a positive correlation between TSH and TV (p < 0.05). There was a significant positive correlation between TV and LH, insulin, HOMA-IR (p < 0.05).

**Conclusion:**

This study showed that TV was increased in patients with insulin resistance but differences in TSH and LH levels may affect TV changes as well.

## INTRODUCTION

Polycystic ovary syndrome (PCOS) is a common endocrine disorder that affects 5-10% of women reproductive age. It is characterized by hyperandrogenism, menstrual irregularity, anovulation, infertility, obesity, early atherosclerosis, and increased cardiovascular risk (1). Recent studies have shown an association between PCOS and thyroid dysfunction, including thyroid volume (TV) changes (2). However, the mechanism of this association remains unclear. In patients with PCOS, central gonadotropin release is changed in pulse frequency and the pulse amplitude of luteinizing hormone (LH) is increased. Increased LH levels are detected in 50% of PCOS patients, but follicle-stimulating hormone (FSH) levels are normal or below normal (1). Thyroid stimulating hormone (TSH), FSH, and LH are accepted as human chorionic gonadotropin (HCG)-like hormones. TSH, FSH, and LH are glycoprotein hormones; they have similar alpha subunits but different beta subunits. Endogenous TSH increases TV (3). It has been shown that both HCG and LH have thyrotropic effects as well (4,5). So, theoretically, FSH and LH changes in PCOS may affect TV. It is known that, hormones as FSH, LH and oestrogen have effects on the pathogenesis of thyroid diseases in women. The difference in thyroid disease incidence between genders may support this effect (6).

Insulin resistance (IR) is one of the characteristic findings in patients with PCOS. Recently, many studies have reported an association between IR and TV, nodule frequency, and thyroid nodule volume (6-8). Moreover, metformin decreased thyroid nodule volume among patients with IR (9). So, the primary objective of this study was to investigate the effect of gonadotropin, sex hormone levels and insulin resistance on thyroid functions and thyroid volume in 69 new diagnosed polycystic ovary syndrome patients and 56 healthy controls female.

## SUBJECTS AND METHODS

This prospective study was conducted between June 2014 and September 2015 in the Department of Endocrinology and Metabolism. It was approved by the local ethical committee (Date: 23.06.2014; Decision Number: 2014/09-08) and written informed consent was obtained from all subjects.

### Subjects and study protocol

Seventy-four patients who were newly diagnosed with PCOS (PCOS group), and 56 healthy females (control group), were included in the study. In the PCOS group, five patients were ruled out due to follow-up failure, so the group proceeded with 69 patients.

For the diagnosis of PCOS, the 2003 Rotterdam criteria were used (10). Being positive for at least two of three criteria (1-oligo and/or anovulation; 2-clinical and/or biochemical hyperandrogenism; and 3-PCOS findings on ultrasound) was accepted as a diagnosis of PCOS, after other possible causes were excluded. All PCOS patients were examined for findings of hyperandrogenism, such as hirsutism, androgenic alopecia, acne, and virilism. Hirsutism was evaluated with the modified Ferriman-Gallwey score (FGS) and a score ≥ eight was accepted as hirsutism (11). Androgenic alopecia was evaluated with the Ludwig score (stage I, II, and III) (12).

Blood pressure was measured in all patients in the right arm after resting in a seated position for five minutes. Height, weight, and body mass index (BMI) values were recorded for all participants. Body mass index was measured as weight/height^2^ (kg/m^2^). Body mass index levels were classified as follows: 18.5-24.9 kg/m^2^ = normal; 25-29.9 kg/m^2^ = overweight; and ≥ 30 kg/m^2^ = obese.

### Exclusion criteria

The following criteria were used to exclude patients from the study: use of drugs containing steroids and/or sex hormones; drugs associated with hirsutism; congenital adrenal hyperplasia or increased 17-alpha-hydroxyprogesterone levels; Cushing syndrome or increased cortisol levels; obesity; prediabetes or diabetes; thyroid disease or thyroid hormone dysfunction; severe iodine deficiency (urine iodine levels < 20 µg/L); and smoking.

Control group participants were selected from patients with regular menstrual cycles, normal androgen levels, and normal hirsutism scores that presented to the endocrinology polyclinic.

Fasting plasma glucose (FPG), lipid profile [triglycerides (TG), low density lipoprotein (LDL), and high density lipoprotein (HDL)], insulin, TSH, free triiodothyronine (fT3), free thyroxin (fT4), anti-thyroglobulin (anti-Tg), anti-thyroid peroxidase (anti-TPO), FSH, LH, estradiol (E2), progesterone, prolactin, cortisol, total testosterone (TT), dehydroepiandrosterone sulfate (DHEAS) and urinary iodine (first morning urine) levels were evaluated in both the PCOS and control groups. Thyroid and pelvic ultrasounds were performed in all participants to assess thyroid nodules, TV, polycystic ovaries, and ovarian volume.

### Biochemical analyses

Blood samples were obtained in 5^th^ day of the menstrual cycle in patients with oligomenorrhea but in patients with amenorrhea blood samples were obtained any day after 8-10 hours of fasting. Blood samples were obtained in sitting position at 8.30 morning from all participants. Normal reference values were as follow; TG (0-150 mg/dL), HDL (26-86 mg/dL), LDL (0-130 mg/dL), TSH (0.4-4.2 uIU/mL), fT3 (1.8-5.2 ng/mL), fT4 (0.8-2.7 ng/dL), thyroglobulin (1.6-59.9 ng/mL), anti-TPO (0-59.9 IU/mL), anti-TG (0-58.5 IU/mL), FSH (1.42-15.4 mIU/mL), LH (1.24-7.8 mIU/mL), TT (15-60 ng/dL), prolactin (3-14.7 ng/mL), E2 (0-750 pg/mL), progesterone (0.2-728.2 nmol/L), DHEAS (35-560 ug/dL), cortisol (5-23 µg/dL) and insulin (6-27 uIU/mL). For urine iodine levels; < 20 µg/L severe iodine deficiency, 20-49 µg/L moderate iodine deficiency, 50-99 µg/L mild iodine deficiency, 100-199 µg/L normal iodine level, 200-299 µg/L increased iodine levels, > 300 µg/L markedly increased iodine levels (13). For urine iodine measurement colorimetric and sandell-kolthoff reaction methods that approved by World Health Organisation (WHO) and International Council for Control of Iodine Deficiency Disorders (ICCIDD) were used (14).

Hormone analyses were carried out using chemiluminescence enzyme immunoassay (Immulite 2000, Diagnostic Products Corporation, Los Angeles, CA, USA, or UniCelDxI 800, Beckman Coulter CA, USA) commercial kits. Glucose levels were measured with glucose oxidase method. Lipid parameters were measured with cholesterol esterase enzymatic assays and Roche T800 Modular analyser (Roche Diagnostic, Turkeys).

OGTT with 75 g glucose were performed in all patients even in normal FPG. After 8-10 hours fasting, fasting glucose and 2^nd^ hour glucose after 75 g glucose were measured. Plasma glucose levels were evaluated according to American Diabetes Association 2013 criteria (15). GDM was diagnosed when any one measurements were higher than normal limits.

Insulin resistance was measured with Homeostatic Model Assessment index (HOMA) [(fasting insulin level [µU/mL] x FPG [mg/dL])/405] and > 2.5 values accepted as IR (16).

1 mg overnight dexamethasone suppression test was performed to all participants to exclude the cushing syndrome and ≤ 1.8 levels was defined as suppressed.

### Pelvic ultrasonography

Ovarian volumes of the patients were evaluated at the day of the blood sampling. Ovarian volumes were evaluated transabdominally with same radiologist and same ultrasound device (Toshiba Medical Systems Corporation, Otawara, Japan) Aplio 400 ultrasound device with 3.5-5.0 Mhz convex probe). Cyst count and dimension were noted. Ellipsoid formula was used to measure the ovary volume (π/6 x transverse diameter x anteroposterior diameter x superoinferior diameter). Ovarian volume under 10 mL was accepted as normal and over 10 mL was accepted as increased over volume.

### Thyroid ultrasonography

Thyroid ultrasonography was performed to all participants and nodule, parenchymal heterogeneity were noted. Grey scale measurements were measured with same endocrinologist and with same device (Logic 5 Doppler system, General electric medical systems, Milwaukee, WI, USA 12 Mhz lineer transduser). Thyroid ultrasonography was performed to participants when they are in supine position, 3 dimensions of the thyroid gland were screened and length, thickness and width of each lobe were measured. The volume of each lob was measured with this formula (17); volume (mL) = π/6 × width × thickness × length.

### Statistical analysis

The SPSS for Windows (ver. 11.0.; Chicago, IL, USA) was used for statistical analysis. The Shapiro–Wilk test was performed to test for a normal distribution. The Independent Simple T-test was used to compare dependent variables that were in accordance with a normal distribution, and the variables were expressed as the mean ± standard deviation. Direct relationship between variations was evaluated with pearson correlation test. P < 0.05 was defined as statistically significant. Direct regression analyze with stepwise method was used to show the effects of insulin, LH and TSH on TV.

## RESULTS

Sixty-nine female who were newly diagnosed with PCOS (age, 24,82 ± 6,17) and 56 healthy female (age, 26,69 ± 5,25) were involved to study.

The demographic and clinic characteristics and radiological parameters of the patients with PCOS and controls are presented in Table 1. There was no significant difference for age, BMI and blood pressure (p > 0.05). FGS and mean ovary volume were significantly higher in PCOS group compared to control group (p < 0.001 and p < 0.001, respectively).

**Table 1:** The demographic and clinic characteristics and radiological parameters of the PCOS patients and controls

Parameters	PCOS group n = 69	Control group n = 56	p
Age (year)	24.82 ± 6.17	26.69 ± 5.25	0.075
BMI (kg/m^2^)	21.86 ± 2.08	21.48 ± 2.16	0.325
Sistolic blood pressure (mmHg)	106.59 ± 10.42	107.69 ± 11.66	0.578
Diastolic blood pressure (mmHg)	66.95 ± 7.96	67.41± 7.97	0.752
Complaint (n/%)			
Hirsutism	51/73.9		
Oligo/amenore	14/20.3	-	-
Acne	2/2.9		
Alopecia	2/2.9		
FGS	11.01 ± 2.74	2.92 ± 2.12	**0.000**
Ultrasonography			
PCO image (n/%)	48/69.56	-	**-**
Mean ovary volume (mL)	10.95 ± 5.45	6.87 ± 1.27	**0.000**

PCOS: polycystic ovary syndrome; BMI: body mass index; FGS: Ferriman-Gallwey score; PCO: polycystic ovary.

Changes in the metabolic and hormonal parameters between PCOS and control group are presented in Table 2. Insulin, HOMA-IR, LH, E2, prolactin, DHEAS and TT levels were significantly higher in PCOS group (p < 0.001, p < 0.001, p < 0.001, p = 0.038, p = 0.012, p = 0.001 and p < 0.001, respectively).

**Table 2:** Changes in the metabolic and hormonal parameters between PCOS and control group

Parameters	PCOS group n = 69	Control group n = 56	p
Glucose (mg/dL)	80.76 ± 8.62	80.62 ± 8,59	0.927
Glucose_2_ (OGTT)	86.49 ± 17.70	87.69 ± 20,27	0.724
LDL (mg/dL)	89.78 ± 25.27	92.15 ± 20,28	0.09
HDL (mg/dL)	53.32 ± 10.13	56.03 ± 10,56	0.146
TG (mg/dL)	81.98 ± 43.32	90.51 ± 41,75	0.268
**Insulin (uIU/mL)**	11.29 ± 6.93	6.28 ± 3,44	**0.000**
**HOMA-IR**	2.29 ± 1.52	1.24 ± 0,69	**0.000**
**LH (mIU/mL)**	12.92 ± 9.16	5.50 ± 2,92	**0.000**
FSH (mIU/mL)	5.80 ± 3.15	6.62 ± 2,04	0.096
**E2 (pg/mL)**	79.07 ± 60.34	59.58 ± 38,13	**0.038**
Progesterone (nmol/L)	1.50 ± 1.71	1.00 ± 1,05	0.059
**DHEAS (ug/dL)**	265.30 ± 118.25	197.81 ± 93,95	**0.001**
**TT (ng/dL)**	50.01 ± 25.28	33.60 ± 13,34	**0.000**
**Prolactin (ng/mL)**	16.64 ± 9.69	12.94 ± 5,28	**0.012**
Cortisol (mcg/dL)	14.16 ± 5.63	14.68 ± 5,69	0.611
**Cortisol (1 mg DST)**	**0.65 ± 0.28**	0.77 ± 0,35	**0.058**

PCOS: polycystic ovary syndrome; OGTT: oral glucose tolerance test; LDL: low density lipoprotein; HDL: high density lipoprotein; TG: triglycerides; HOMA-IR: homeostasis model assessment-insulin resistance; LH: luteinizing hormone; FSH: follicle-stimulating hormone; E2: estradiol; DHEAS: dehydroepiandrosterone sulfate; TT: total testosterone; DST: dexamethasone suppression test.

There was no significant difference for mean TSH, fT4, thyroglobulin, urinary iodine, prevalence of nodule and thyroid antibody (p > 0.05). Thyroid volume was significantly higher in PCOS group (p = 0.033) (Table 3).

**Table 3:** Changes in the thyroid spesific characteristics between PCOS and control group

Parameters	PCOS group n = 69	Control group n = 56	p
TSH (uIU/mL)	2.49 ± 1.00	2.29 ± 1.03	0.296
fT4 (ng/dL)	1.11 ± 0.12	1.14 ± 0.13	0.09
Anti-TPO positivity (n/%)	6/ 8.7	7/12.5	0.343
Anti-TG positivity (n/%)	7/10.1	4/7.14	0.397
Thyroglobulin (ng/mL)	13.45 ± 9.58	10.94 ± 7.87	0.118
Urinary iodine (µg/L)	97.84 ± 38.30	91.83 ± 37.42	0.381
Thyroid nodule (n/%)			
NG	7/10.1	6/10.71	0.446
MNG	8/11.6	3/5.35	0.371
Thyroid volume (mL)	12.68 ± 3.07	11.45 ± 3.27	0.033

PCOS: polycystic ovary syndrome; TSH: thyroid stimulating hormone; fT4: free thyroxin; anti-TPO: anti-thyroid peroxidase; anti-Tg: anti-thyroglobulin; NG: nodular goiter; MNG: multinodular goiter.

Patients with PCOS were divided, according to HOMA-IR, into 2 subgroups: non-insulin resistant (NIR-PCOS), and insulin resistant PCOS (IR-PCOS). Thyroid volume was statistically significant higher in IR-PCOS group (p < 0.001). There was no significant difference for TSH, fT4, thyroglobulin and urinary iodine (p > 0.05) (Table 4).

**Table 4:** Changes in the thyroid spesific characteristics between IR-PCOS and NIR-PCOS

	IR-PCOS n = 25	NIR-PCOS n = 44	p
TSH (uIU/mL)	2.69 ± 1.07	2.37 ± 0.96	0.213
fT4 (ng/dL)	1.10 ± 0.13	1.11 ± 0.12	0.631
Thyroglobulin (ng/mL)	11.37 ± 8.92	14.63 ± 9.84	0.177
Urinary iodine (µg/L)	94.96 ± 41.07	99.47 ± 37.03	0.641
**Thyroid volume (mL)**	**14.43 ± 2.91**	11.68 ± 2.72	**0.000**

IR-PCOS: insulin resistant polycystic ovary syndrome; NIR-PCOS: non-insulin resistant polycystic ovary syndrome; TSH: thyroid stimulating hormone; fT4: free thyroxin.

There was negative correlation between TSH levels and E2 (r = -0.261, p = 0.031), and a positive correlation with TSH and TV (r = 0.319, p = 0.008). There was no correlation between TSH and BMI, fT4, urinary iodine, FSH, LH, thyroglobulin, TT, E2, prolactin, glucose, insulin, HOMA-IR and ovary volume (p > 0.05). Additionally, there was a positive correlation between TV and LH, insulin and HOMA-IR (r = 0.177, p = 0.048; r = 0.375, p = 0.001 and r = 0.361, p = 0.002, respectively), but there was no correlation between TV and BMI, fT4, urinary iodine, FSH, thyroglobulin, TT, E2, prolactin, glucose levels and ovary volume (p > 0.05). Direct regression analyze with stepwise method revealed that insulin, LH and TSH may explain the 88,5% of the change on TV and the model to assess the change was as follow; “TV = 2.895 x TSH + 0.318 x insulin + 0.130 x LH”.

## DISCUSSION

Thyroid disorders and PCOS are two of the most common endocrine disorders in the general population (18,19). Insulin resistance and gonadotropin axis changes are thought to contribute to the pathogenesis of PCOS (1), however, there is limited data about the effect of these changes on thyroid hormones and TV (2).

Thyroid diseases are seen more frequently in women, so in this regard, FSH, LH, and E2 are important to consider (19). The research about the effect of E2 on TV is conflicting. It has been shown that increased E2 levels have mitogenic and proliferative effects on thyroid cells (20,21), but some studies have concluded that chronic estrogen treatment may decrease TV (22). There is a hyperestrogenic state in PCOS (23), but data about the effects of E2 levels on PCOS patients are contradictory. Cakir and cols. (24) reported that E2 levels were significantly lower in PCOS patients and E2 levels were not associated with TV. In this study, E2 levels were significantly higher when compared with the control group, and there was a significant negative correlation between E2 and TSH, but no significant correlation between E2 and TV. The lack of expected effects of E2 on TV may be due to the negative effect of E2 on TSH.

TSH, FSH, and LH are glycoprotein hormones; they have similar alpha subunits but different beta subunits. Endogenous TSH increases TV (3,25,26). It has been shown that HCG increases TSH receptor expression, thyroid hormone secretion, iodine uptake, organification, and DNA synthesis in both rat and human thyrocyte cultures (27,28). LH is more potent than HCG for binding TSH receptors and increasing adenylate cyclase (5,29). In PCOS patients, LH levels are higher than in controls, even in the follicular phase (30,31). Dewailly and cols. (32) reported serum LH and the LH/FSH ratio in patients with PCOS to be higher compared with the control group. Jamil and cols. (33) reported that LH levels were higher in the PCOS group. A positive association between LH and TV has been reported as well (24). In this study, LH levels were significantly higher in the PCOS group, and there was a positive correlation between LH and TV. This suggests that changes in LH levels may lead to thyrotrophic effects and cause changes in TV.

Thyroid volume is associated with hyperinsulinemia (34). Insulin receptor expression is increased in thyroid cells of diabetic rats (35). Furthermore, there is evidence showing that insulin increases thyroid proliferation (36). Therefore, high circulating insulin levels may increase thyroid proliferation and cause thyroid nodule formation and an increase in TV. Rezzonico and cols. (7) reported an association between thyroid nodularity and IR. However, the data about thyroid nodularity and TV in IR-PCOS patients is contradictory. Several studies have reported a positive association between IR, TV, and nodularity (2), but contrary results have also been reported (24,37). In the present study, both insulin levels and IR were higher in the PCOS group. TV was significantly higher in IR-PCOS patients compared with NIR-PCOS patients. Moreover, a positive association between insulin levels and HOMA-IR with TV was detected. This suggests that IR may be one of the causes of TV changes in PCOS patients.

A positive association between TSH levels and IR in PCOS patients has been reported (2). However, Cakir and cols. (24) found no significant difference in TV between IR-PCOS patients and NIR-PCOS patients. In this study, there was no significant difference in TSH and fT4 levels between both the PCOS and the control group and in the NIR-PCOS and IR-PCOS groups. However, in the PCOS group, TV was significantly higher than in the control group and there was a positive correlation between TSH and TV. Unlike other studies, in this study, the iodine status of the participants was evaluated and similar iodine status, both in the PCOS and control group (mild iodine deficiency), was found. This may be the reason for similar TSH and fT4 levels between the control and PCOS patients. However, it may be proposed that TSH levels affect TV because of a significant association between TSH and TV.

TV is influenced by urine iodine status, age, gender, height, weight, BMI, and body surface area (38). In this study, age, BMI, and iodine status were similar, so it is concluded that these factors did not affect TV in the study participants.

It has been shown that autoimmune thyroid diseases are more frequent in PCOS patients (39,40), but no difference in thyroid autoimmunity between PCOS patients and healthy controls was shown as well (41). Hyperestrogenism is thought to be responsible for more frequent autoimmune diseases in women compared to men (42). Janssen and cols. (40) demonstrated increased thyroid autoantibodies, TV and thyroid hypoechogenicity in PCOS patients. Garelli and cols. (43) reported that anti-TPO positivity was 27% in the PCOS group and 8% in the control group. But Anaforoglu and cols. (6) claim that PCOS alone is not associated with thyroid disease, metabolic syndrome components may be associated with thyroid autoimmunity. In this study, there was no significant difference between groups according thyroid antibodies. This may be due to small number of patients with positive thyroid antibodies.

This study demonstrates that TV is increased in PCOS patients and this change is especially due to hyperinsulinemia and IR since NRI-PCOS have the same TV as controls. This study also showed that TSH and LH levels may be independently responsible for TV increases in PCOS patients. However further more detailed studies are needed to address this issue.
